# Pain assessment of a new bloodletting device: A study protocol for a randomized, controlled, double-blind, matched-paired clinical trial

**DOI:** 10.1097/MD.0000000000018705

**Published:** 2020-01-31

**Authors:** Hwa Yeon Ryu, Jae Hui Kang

**Affiliations:** a Department of Acupuncture and Moxibustion Medicine, College of Korean Medicine, Daejeon University; b Department of Acupuncture and Moxibustion Medicine, Cheonan Korean Medicine Hospital of Daejeon University, Daejeon, South Korea.

**Keywords:** bloodletting device, protocol, randomized controlled trial

## Abstract

**Background:**

The bloodletting device has been used by many institutions for about 100 years. Many patients feel fear from the pain caused by applying the bloodletting device for treatment. We used bloodletting device using the principle of “prestimulation neurodisturbance,” which can mask the subject undetectable for pain. In this study, we will investigate pain of bloodletting device during blood collection and will identify the safety of the device.

**Methods:**

This study will be a randomized, controlled, double-blind, and matched-paired-designed clinical trial. Four groups, RTLC, LTRC, RCLT, and LCRT (T = test device, C = control device, L = left, R = right), will be randomly allocated. Total duration of the clinical trial will be 3 months. The subjects will be performed from 1 to 3 times only on the day of the procedure. The primary outcomes will be measured using pain visual analog scale score and the secondary outcomes will include verbal rating scale and the time at which the pain disappears after blood collection (second), the total number of “nonbleeding” cases and subjects, the number of “blood collection failure” and subjects, the presence of “delayed hemostasis,” and the number of subjects. Repeated-measure analysis will be used to measure primary efficacy based on full analysis set.

**Discussion:**

This study has limited inclusion and exclusion criteria and a well-controlled intervention, and it will be the first randomized controlled trial to investigate pain of bloodletting device using the principle of “prestimulation neurodisturbance.” This study provides insights into the underlying mechanisms of the pain-reducing effect of the developed bloodletting device and will lay the groundwork for further studies.

## 1 Introduction

The bloodletting device has been used by many institutions for about 100 years but mostly by hospitals that practice western medicine and Korean medicine. Bloodletting devices are used for various purposes, such as blood collection for examinations or blood collection to relieve muscle tension. In our Korean medicine hospital, many patients fear the pain caused by applying the bloodletting device for the treatment of a medical condition.

Improvements have been made in the mechanical elements of existing bloodletting devices, such as thinning, smoothing, and shortening the needle. However, since the thickness of the skin is different for each person, there is a disadvantage that the depth of the needle cannot be precisely adjusted.

In the present study, the principle of “prestimulation neurodisturbance” was used; that is, if the area is first stimulated with another stimulus before the needle enters the skin, the nerves in the femoral cortex are disturbed. When the nerve is disturbed, the needle pierces the skin quickly and falls out, masking the pain.^[1]^ Therefore, we designed a study protocol to investigate the pain associated with use of a new bloodletting device by using a randomized, controlled, double-blind, and matched-paired-designed clinical trial.

## 2 Materials and methods

### 2.1 Study design

This randomized, controlled, double-blind, matched-paired clinical trial will compare the pain associated with use of 1 bloodletting device (ROAH-L-NO1) to that associated with a control device (LANZO 1.5). One hundred outpatients will be recruited from the Daejeon University Cheonan Korean Medicine Hospital through advertisements posted on bulletin boards in hospitals. Recruitment commenced in October 2019, and the trial is expected to end in December 2019. One hundred patients who meet the eligibility criteria for this study will be randomly assigned to groups A, B, C, or D. The intervention will begin on the day of the screening visit. Depending on the study group, 2 procedures will be performed, 1 for each participant's hand, in the order of the right and left hand labeled on the bloodletting devices according to the randomization code. The total duration of the clinical trial will be 3 months.

### 2.2 Inclusion and exclusion criteria

The inclusion criteria are as follows: age, 19 to 64 years; ability to collect blood on the same finger of both hands; and patients who can communicate clearly and express their pain. The exclusion criteria are as follows: patients with blood clotting disorders, such as hemophilia or leukemia; those with structural disorders, such as defects or deformations of the blood collection area; patients who should avoid blood collection because of wounds and skin diseases; and those with sensory disorders in the area of blood collection, such as hemiplegia or peripheral nerve damage.

### 2.3 Withdrawal and dropout

If the participant does not meet the inclusion or exclusion criteria or withdraws his/her consent, or if the participant's continued participation is judged as inappropriate, or if 3 instances of nonbleeding occurs on the same finger of a participant, he/she will be excluded from the study. The researchers will record the reason for any interruption in the intervention and whether each participant completed the study.

### 2.4 Sample size calculation

The basis for calculating the subjects of this study was derived from the results of previous studies by Baxter et al.^[2,3]^Table 1 summarizes the evaluation results of those studies to estimate the effect size on the primary efficacy endpoint, visual analog scale (VAS) (100 mm).

**Table 1:**
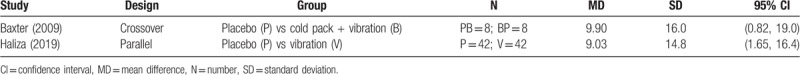
Evaluation results of the 2 studies (Baxter and Haliza).

The results were combined using the method described by Curtin, and the estimated magnitude of Buzzy effect on pain reduction during intravenous blood collection is summarized in Table 2.^[4–6]^

**Table 2:**
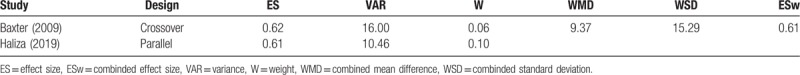
Estimated magnitude of Buzzy effect on pain reduction.

Because the present study will proceed to the point of blood collection, not only the pain intensity is weaker than the previous study, but the duration and intensity of vibration are shorter or weaker than that of Buzzy effect. Therefore, the effect size on the pain reduction of the test device is thought to be smaller than that of Buzzy effect. In the design of the clinical trial, 4 sequences will be assigned, but since the 2 treatments and 2 periods are basically 2 × 2 cross designs, the formula for calculating the sample size corresponding to the design will be applied mutatis mutandis. Assuming that the effect size of the study device is n = (0.61 ÷ 2) ÷ 3,” the sample size (class 1 error *a* = 0.05; power 1 − *b* = 0.8) to be assigned to each device sequence will be as follows: 
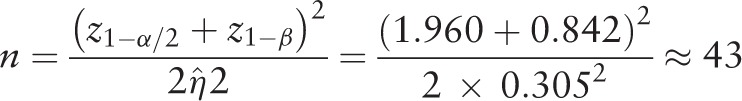


Forty-three participants will be required for each treatment group. In addition, considering a 15% dropout rate, the number of participants required per treatment group will be 50. One hundred participants will be calculated as the total number of participants in this study.

### 2.5 Randomization and blinding procedures

There will be 4 assignments according to the order of the bloodletting devices (T = test device, C = control device) and blood collection location (L = left, R = right): RTLC (group A), LTRC (group B), RCLT (group C), and LCRT (group D). Balanced block randomization will be used to evenly assign the participants to each group. The process will be as follows:

1. number the assignment for each group (A = group A, B = group B, C = group C, D = group D),
2. randomly select 1 block size among 4, 8, 12, and 16,
3. randomly select 1 sequence that can be allocated to 4 groups evenly for each block size,
4. assign participants to the selected sequence, and
5. repeat steps 2 to 4 for 100 participants.

Participants who meet all registration criteria will be randomly assigned to a randomization identification code (e.g., BCU-R-001, BCU-R-002, …, BCU-R-036) in the order generated by a computer randomized program. The randomization identification code indicates the type of bloodletting device and the order of the left and right hands. Additionally, the participants and the assessors who will be collecting the data will be blinded to the group allocation.

Information of the intervention assignment will be stored in the 3rd statistical department. The randomization code will be placed in an opaque envelope and stored in the hospital. With the exception of disclosure to individual patients in emergency situations, randomization, and blinding will not be disclosed to researchers until the end of the trial.

### 2.6 Intervention

The test device (ROAH-L-NO1) will be manufactured by Roahmed Medical Device Co. (Changwon, Republic of Korea), and the control device (LANZO 1.5) will be manufactured by GMMC Medical Device Co (Seoul, Republic of Korea). Both devices will be applied to the participants’ 3rd finger.

All eligible participants will receive a treatment according to the allocated group, RTLC, LTRC, RCLT, or LCRT, on the day of the screening visit. To maintain the double-blinded nature of the study, during the procedure, a screen will be placed between the participant and the operator. The operator will use the test and control devices on the participants on the outside of the screen, and participants will be indistinguishable to which hand the test device is assigned. The operator will practice using the randomized device on the 3rd finger of 1 hand of the participant. If blood is collected to the extent that the blood glucose level can be measured, it will be determined to be successful, and the 1st measurement will be completed. If no blood is collected, the participant will be checked to confirm no bleeding (“nonbleeding”), and treatment will be applied again after 10 minutes to determine whether blood collection is successful. When the total number of instances of nonbleeding is 3, no further procedure will be recorded, and “blood collection failure” will be recorded. The operator will check the participants’ state of hemostasis after bleeding, and if hemostasis does not occur within 3 minutes (“delayed hemostasis”), the participant will be checked again for complete hemostasis. If hemostasis does not occur for more than 10 minutes, the operator will check the participant for hemostasis abnormalities, and the operator will stop the clinical trial and seek medical attention for the participant. The secondary measurement will be performed 10 minutes after the primary measurement in the same way on the same area of the participants’ opposite hand. Participants will undergo the intervention from 1 to 3 times only on the day of the procedure.

### 2.7 Outcome measures

The primary outcome will be the VAS score, which will be assessed upon successful blood collection. The secondary outcomes will be the verbal rating scale (VRS) score and the time at which pain disappears after blood collection (second), which will be assessed upon successful blood collection. In addition, the total number of nonbleeding cases, number of blood collection failures, number of cases of delayed hemostasis, and number of participants will be counted. Participants’ data will be anonymized and coded by a specific program.

### 2.8 Data collection and monitoring

During the screening period, participants will complete their sociodemographic characteristics collected by the questionnaire. Personal information and data collected during the screening period will be shared and managed by the hospital. The monitoring of data and research performances will be conducted regularly by researchers from the Cheonan Korean Medicine Hospital of Daejeon University (Cheonan, Republic of Korea). The final trial dataset will be accessible to statisticians and the principal investigator (PI).

### 2.9 Statistical analysis

Statistical analysis will be primarily based on the principle of the full analysis set. Missing values will be analyzed by the last observation carried forward method. For each of the 2 devices, the VAS score and time at which the participant's pain disappears will be measured, and descriptive statistics will be presented as a mean, standard deviation, median, and minimum and maximum values. The analysis method for evaluation of the variables of efficacy will involve confirmation of the normality of the values and then comparison using the paired *t* test or Wilcoxon signed-rank test. The McNemar test will be used to analyze the VRS score, total number of nonbleeding cases, number of blood collection failures, number of cases of delayed hemostasis, and number of participants. The significance level will be set at *P* < .05 and the confidence interval at 95%. All statistical analyses will be performed using SPSS Statistics for Windows version 20.0 (IBM Corp, Armonk, NY).

If necessary, subgroup analyses of outcome variables, such as the demographic variables measured at baseline (e.g., sex, age, duration of illness, treatment expectation) can be performed. Safety assessments will be performed primarily by analyzing the frequency of adverse events and serious adverse events (SAEs) that researchers suspect to be related to treatment. The collected safety data will be summarized appropriately. All adverse events that occur will be listed with a detailed description. All SAEs will be recorded descriptively. The adverse events will be collected through patient symptom reporting and researcher observation. The frequency of each adverse event associated with or unrelated to the intervention will be recorded and analyzed using descriptive statistics. There is no intermediate analysis of this study, and the final decision regarding the completion of the clinical trial will be made by the PI.

### 2.10 Safety and analysis

Participants will be monitored for undesirable, unintended symptoms, signs, and illnesses. The number and percentage of participants who experience at least 1 adverse event will be calculated and tested using the Chi-squared test or Fisher exact test.

### 2.11 Ethics

This study is designed based on the Helsinki Declaration and the Korean Clinical Practice Guidelines, and it has been approved by the Korean Institutional Review Board of DUCKMH (approval number DJUMC-2019-MD-01-1). This study protocol is registered with CRIS (CRIS-KCT0004478). All participants will receive a full written explanation of the study's protocol and an informed consent form. Participants may be required to quit the study in case of SAEs or adverse drug reaction, and then they will be reported to the institutional review board of the hospital. All participants of this study will be allowed to withdraw their consent at any time for any reason or discontinue their participation on a voluntary basis.

## 3 Discussion

The bloodletting device is a medical device used for medical examination by a medical institution or for diabetic patients to measure blood glucose levels. Diabetic patients are the second most common group after medical institutions for whom the bloodletting device is used. According to statistics released by the World Health Organization, the number of diabetic patients worldwide reached 422 million in 2014, with a 7% to 8% increase in the disease annually.^[7–10]^ This group of patients draws blood for themselves, 2 to 3 times a day, to measure their blood glucose levels. Pain during blood collection has caused about 50% of diabetic patients to stop monitoring their own blood glucose levels.

Human skin is classified into the epidermis, dermis, and subcutaneous tissue. For blood collection, the needle only needs to enter the capillaries, that is, just before the subcutaneous tissue.^[11]^ If the needle penetrates too deep, it causes severe pain, so it is usually necessary to reduce the pain by adjusting the depth of the needle. However, all pain appears when the stimulus is applied to the skin and the stimulus signal is transmitted to the femoral cortex. Therefore, no matter how short the needle is, if the needle enters the skin directly, the pain is felt by the patient and a true painless effect cannot be obtained.

For this study, we prepared a bloodletting device using the principle of prestimulation neurodisturbance and planned a randomized clinical trial to evaluate pain during blood collection. However, bias may occur because this trial will be conducted without a pilot study. Nevertheless, this study may provide insight into the underlying mechanisms of the pain-reducing effect of the developed bloodletting device, and can lay the groundwork for further research on the development of painless bloodletting devices.

## Author contributions

JHK designed, administrated and supervised the study protocol and critically revised the manuscript. HYR developed the protocol, drafted the manuscript. Both authors have read and approved the final manuscript.

Hwa Yeon Ryu orcid: 0000-0002-9468-1227.
